# Effects of Maturity and Thermal Treatment on Phenolic Profiles and In Vitro Health-Related Properties of Sacha Inchi Leaves

**DOI:** 10.3390/plants11111515

**Published:** 2022-06-05

**Authors:** Suwapat Kittibunchakul, Chatrapa Hudthagosol, Promluck Sanporkha, Suwimol Sapwarobol, Uthaiwan Suttisansanee, Yuraporn Sahasakul

**Affiliations:** 1 Food and Nutrition Academic and Research Cluster, Institute of Nutrition, Mahidol University, Salaya, Phuttamonthon, Nakhon Pathom 73170, Thailand; suwapat.kit@mahidol.ac.th (S.K.); uthaiwan.sut@mahidol.ac.th (U.S.); 2 Faculty of Public Health, Mahidol University, Ratchathewi, Bangkok 10400, Thailand; chatrapa.hud@mahidol.ac.th (C.H.); promluck.san@mahidol.ac.th (P.S.); 3 Faculty of Allied Health Sciences, Chulalongkorn University, Pathumwan, Bangkok 10330, Thailand; suwimol.sa@chula.ac.th

**Keywords:** *Plukenetia volubilis* L., tea, young leaves, old leaves, antioxidant activity, enzyme inhibition, glycation, non-communicable diseases

## Abstract

Sacha inchi (*Plukenetia volubilis* L.) has been adopted as a novel economic crop with well-studied nutritional and bioactive benefits for human health. Sacha inchi seeds and oil have high commercial value but scant research has focused on its leaves. This study investigated and compared phenolic compositions, antioxidant potentials and in vitro health-related properties of both young and mature sacha inchi leaves after freeze-drying and oven-drying processes. Results showed that *p*-coumaric acid, 4-hydroxybenzoic acid, ferulic acid and gallic acid were predominantly detected in both young and mature leaves that also exhibited similar total phenolic contents (TPCs), while higher TPCs were detected in freeze-dried than in oven-dried leaves. Mature leaves exhibited higher antioxidant potential than young leaves after freeze-drying, while the opposite results were observed for oven-drying. Overall in vitro health-related activities were higher in mature leaves compared to young leaves regardless of the drying process. Knowledge gained from this study can be used to encourage prospective utilization of sacha inchi leaves as a source of health-promoting compounds. This, in turn, will increase the commercial value of the leaves and provide a wider market variety of sacha inchi products.

## 1. Introduction

Sacha inchi (*Plukenetia volubilis* L.), also known as Inca peanut, sacha peanut or mountain peanut is native to the Peruvian Amazon. It has long been a staple crop in the diets of Latin American populations, and also a herbal remedy used widely by indigenous locals in Peru [1,2]. Traditionally, seeds of sacha inchi are consumed roasted, milled to flour, or pressed to obtain a cooking oil that can also be employed in the treatment of muscular pain and rheumatic disease [1]. Young leaves are primarily eaten raw in salads, or dried and used to brew tea [1,3]. Sacha inchi has attracted considerable attention in recent years as a novel economic crop with great potential for food, pharmaceutical, and nutraceutical applications [2,4]. Consequently, the plant has now spread from the Amazon to other global regions such as China, Thailand and Vietnam [5]. The growing interest in sacha inchi is due to its competitive agronomic properties, as well as the excellent nutritional and bioactive compositions that benefit human health [1,5]. 

Sacha inchi contains several types of valuable biomolecules whose presence is distributed across the edible parts of the plant. The seeds are rich in lipids (35–60%, comprising mainly polyunsaturated fatty acids) and protein (27–33%, including essential amino acids) [6,7]. Seed oil is known to contain several phytochemicals, e.g., tocopherols, phytosterols, carotenoids and polyphenols [2,3,6]. Husks and shells are nutrient-rich, especially the fiber and composed of health-promoting phenolics [8]. These sacha inchi parts have been previously reported to possess suppressive effects on some non-communicable diseases (NCDs) such as glycation reaction, hyperpigmentation and oxidative stress-related disorders [1,5,8,9]. Extensive research has been conducted on different plant parts of sacha inchi but few studies have focused on the leaves. Fresh leaves (unknown maturity stage) of sacha inchi contain terpenoids, saponins, phenolics, and other substances responsible for antioxidative and antiproliferative effects [10]. Antioxidant activities of leaves (unknown maturity stage) that underwent air drying were observed in vitro [11]. Roasting at 60 °C increased the antioxidant potential of sacha inchi leaves (unknown maturity stage), compared to fresh and air-dried leaves [12]. Moreover, an in vivo study found that hot-water extracted leaves (unknown maturity stage) improved glycemic control and gut microbiota composition in mice, with possible potential as a functional drink [13]. However, how leaf maturity impacts the health-related properties of sacha inchi has not been reported. 

This study investigated and compared phenolic compositions, antioxidant, anti-glycation and enzyme inhibitory activities of young and mature sacha inchi leaves. The leaves were dried with and without thermal application to observe the impact of the drying processes. Hot-water extraction of the dried leaves was conducted as infusions. Enzyme inhibitory assays were performed using the key enzymes involved in type II diabetes, obesity and Alzheimer’s disease (AD). To the best of our knowledge, this is the first study demonstrating the effects of leaf maturity and the drying process on the phenolic profiles and bioactivities of sacha inchi leaves. Knowledge gained from this study can support further research on the health benefits of sacha inchi leaves using cell culture models or in vivo investigations, thus opening up the feasibility of leaf applications as a resource for biologically active compounds. Our results can be used to expand the industrial applications of this plant as a functional ingredient to develop healthy food products and dietary supplements following toxicological evaluation, thereby increasing the commercial value of sacha inchi leaves and also providing a wider market variety of sacha inchi products. 

## 2. Results

### 2.1. Phenolic Compositions

Results obtained from high-performance liquid chromatography (HPLC) suggested that young and mature leaves of sacha inchi shared the same phenolic profile consisting of 11 phenolics, including three flavonoids (kaempferol, apigenin and isorhamnetin), and eight phenolic acids (gallic acid, vanillic acid, 4-hydroxybenzoic acid, caffeic acid, *p*-coumaric acid, syringic acid, sinapic acid and ferulic acid) (Table 1 and Figure S1). Among the flavonoids, kaempferol was abundantly detected at 7.1- to 9.9-fold higher than the others. *p*-Coumaric acid, ferulic acid, 4-hydroxybenzoic acid and gallic acid were the predominant phenolic acids (up to 59.4-, 57.3-, 50.0- and 28.6-fold, respectively, higher than the other phenolic acids) detected in both young and mature leaves. All samples contained only minute amounts of apigenin, isorhamnetin, vanillic acid and syringic acid at less than 6 mg/100 g dry weight (DW).

Comparing the drying processes of sacha inchi leaves at the same maturity stage, freeze-dried young leaves exhibited 1.2–2.3-fold higher flavonoids and 1.1–3.2-fold higher phenolic acids than oven-dried young leaves, with the exception of syringic acid and ferulic acid, which exhibited insignificant amounts. Likewise, freeze-dried mature leaves exhibited 1.2–2.6-fold higher flavonoids and 1.1–2.9-fold higher phenolic acids than oven-dried mature leaves, with the exception of gallic acid.

Comparing the maturity stages of sacha inchi leaves prepared under the same drying process, freeze-dried young leaves exhibited 2-fold higher gallic acid content and 1.5-fold higher 4-hydroxybenzoic acid than their corresponding mature leaves. However, the latter exhibited 1.8-fold higher isorhamnetin content than the former, while other phenolics were within 20% between the maturity stages. Young samples of oven-dried leaves exhibited 1.6-fold higher isorhamnetin, gallic acid and sinapic acid contents than mature leaves, and the latter exhibited 1.7-fold higher apigenin content than the former. Other phenolics were within 25% between the maturity stages. Overall, young leaves exhibited predominantly 4-hydroxybenzoic acid, ferulic acid, *p*-coumaric acid and gallic acid (accounting for >12% of the total detected phenolics), while mature leaves possessed major phenolics as 4-hydroxybenzoic acid, ferulic acid and *p*-coumaric acid (accounting for >15% of the total detected phenolics).

Results obtained from the Folin–Ciocalteu assay indicated that freeze-dried samples exhibited 1.6-fold higher total phenolic content (TPC) than their corresponding oven-dried samples. This result was observed in both young and mature leaves. However, mature leaves exhibited slightly higher TPCs than their corresponding young leaves under the same drying process.

### 2.2. Antioxidant Activities

The in vitro abilities to resist oxidation were investigated by 2,2-diphenyl-1-picrylhydrazyl (DPPH) radical scavenging, ferric ion reducing antioxidant power (FRAP) and oxygen radical absorbance capacity (ORAC) assays (Table 2). The DPPH radical scavenging activities of infusions prepared from all dried leaves were comparable; however, the extract of oven-dried young leaves possessed significantly higher DPPH radical-scavenging activity compared to its freeze-dried counterpart. Radical-scavenging activities ranged from 0.049–0.051 µmol Trolox equivalent (TE)/g DW, with the extract of oven-dried mature leaves exhibiting the highest activity. A different trend was observed in the FRAP and ORAC assays, in which the infusion from oven-dried young leaves exhibited 1.1–1.2-fold higher antioxidant activities than the corresponding freeze-dried leaves at the same maturity stages. Opposite results were observed for the infusions from mature leaves. Insignificantly different FRAP activities were observed between the infusions prepared from mature leaves after the freeze-drying and oven-drying processes but ORAC activity in freeze-dried leaves was 1.1-fold higher than in oven-dried leaves. Comparing maturity stages, the infusions of mature leaves prepared by freeze-drying exhibited 1.1–1.2-fold higher FRAP and ORAC activities than the corresponding young leaves. By contrast, infusions of young leaves prepared by oven-drying exhibited up to 1.1-fold higher antioxidant activities than the corresponding mature leaves.

### 2.3. Enzyme Inhibitory Activities

The in vitro bioactivities of young and mature leaf infusions prepared from the freeze-dried and oven-dried processes were further investigated regarding inhibitions of the key enzymes relevant to some NCDs. These key enzymes can control the occurrence of type II diabetes (α-amylase and α-glucosidase), obesity (lipase) and AD (acetylcholinesterase (AChE), butyrylcholinesterase (BChE) and β-secretase (BACE-1)) (Table 3). The non-enzymatic glycation reaction was also examined as an anti-aging property of sacha inchi leaf infusions (Table 3).

Inhibition of α-amylase and α-glucosidase can retard carbohydrate degradation, slow down the release of sugar, and consequently lead to reduced glucose absorption and low serum sugar levels [14]. The inhibitory activities against α-amylase shown by all leaf infusions were 37.13–41.62% using an extract concentration of 2.5 mg/mL. Considering these inhibitory activities, the half-maximal inhibitory concentration (IC_50_) was determined to be > 2.5 mg/mL. Results also suggested that infusions of oven-dried young leaves demonstrated a significantly higher α-amylase inhibition (~1.1-fold) than their freeze-dried counterparts, while insignificantly different inhibitions were observed in mature leaves prepared by the different drying processes. Regardless of maturity stage, no significant differences in α-amylase inhibition were observed in leaf infusions prepared by both drying processes. For α-glucosidase inhibition, inhibitory activities of all leaf infusions ranged 8.11–11.81% inhibition using concentrations of 2.5 mg/mL. Due to low inhibitory activities, the IC_50_ of the extracts on this enzyme was determined to be >2.5 mg/mL. Infusions of freeze-dried leaves exhibited significantly higher (~1.2-fold) α-glucosidase inhibition than oven-dried samples regardless of maturity stage, while infusions of mature leaves exhibited 1.2-fold higher α-glucosidase inhibitory activities than young leaves prepared under the same drying process. 

Inhibition of the lipid-degrading enzyme lipase can suppress obesity by lowering fat absorption [15]. Results indicated that all leaf infusions retarded lipase activity by 9.43–9.80% using extract concentration of 2.0 mg/mL regardless of maturity stage and drying process. The IC_50_ of the extracts on this enzyme was, thus, >2.0 mg/mL.

Inhibition of the two cholinesterases (AChE and BChE) and BACE-1 can be drug-targeted to prevent and ameliorate AD [16]. Cholinesterases can hydrolyze acetylcholine, the neurotransmitter synthesized and used by cholinergic neurons. The depletion of acetylcholine results in attention and memory deficits, eventually leading to AD distribution. Likewise, BACE-1 is involved in the cleavage of amyloid precursor protein (APP) to generate β-amyloid peptides, resulting in the accumulation of amyloid plaques, the pathological hallmark of AD that accumulate in the brain. Results suggested that all leaf infusions exhibited AChE and BChE inhibitions ranging between 4.15–14.64% and 20.88–30.09%, respectively, using leaf infusion concentration of 2.0 mg/mL. The IC_50_ of the extracts on these enzymes was considered as >2.0 mg/mL. For AChE inhibition, leaf infusions prepared by oven-drying exhibited 2.6–3.4-fold higher inhibitory activities than by freeze-drying regardless of maturity stages. By contrast, leaf infusions prepared by freeze-drying exhibited 1.2-fold higher BChE inhibition than by oven-drying. No significant difference in AChE inhibition was observed between infusions of young and mature leaves prepared by the freeze-drying process; however, infusions of oven-dried mature leaves exhibited 1.3-fold higher inhibition than young leaves prepared under the same drying process. The opposite results were observed for BChE inhibition, in which infusions of mature leaves exhibited 1.2-fold higher inhibitory activities than young leaves regardless of the drying process. However, BACE-1 inhibitory activity was not detected in all leaf infusions at concentrations of 2 mg/mL.

In vitro inhibitions of non-enzymatic glycations by sacha inchi leaf infusions ranged between 78.3–92.98% and 81.35–90.51% induced by glucose and methylglyoxal (MG), respectively, using a concentration of 2.5 mg/mL. Thus, the IC_50_ of the extracts was considered as <2.5 mg/mL. Comparing the drying processes, infusions of oven-dried leaf extracts exhibited up to 1.1-fold higher inhibitory activities against D-glucose- or MG-induced glycation than leaf extracts from the freeze-dried process regardless of maturity stage. Likewise, when considering leaf maturity, the inhibitory activities against D-glucose- or MG-induced glycation were significantly higher (up to 1.1-fold) in infusions of mature leaves compared with young leaves.

## 3. Discussion

Sacha inchi products are becoming increasingly popular in the global market. Products from seeds and oil, such as roasted and seasoned seeds, seed protein powder, gourmet oil and encapsulated oil supplements are now industrialized [1]. Interestingly, young leaves are only sold locally in dried form as herbal tea, while the utilization and commercialization of mature leaves have not been documented. Previous studies suggested that sacha inchi leaves might be a good source of bioactive compounds [10], while dried leaves exhibited antioxidant potential in vitro [11,12]. Nevertheless, the available information is limited, and the effects of leaf maturity on the health-related properties of sacha inchi leaves have not been reported. Here, we investigated and compared the phenolic compositions, antioxidant activities and enzyme inhibitory activities of young and mature sacha inchi leaves that underwent two drying processes as freeze-drying at −50 °C, 0.086 mbar for 72 h, and oven-drying at 60 °C for 5 h. Research on the changes in bioactivities due to leaf maturation and processing can lead to future food and pharmaceutical applications via proper harvesting and handling of the leaves. Fresh leaves of sacha inchi were excluded as their analysis was beyond the scope of this study.

Previous reports have investigated the presence of phenolics in sacha inchi leaves [10,12] but phenolic profiles of the leaves have not been characterized. In this study, eleven phenolics in young and mature sacha inchi leaves that underwent drying were identified by HPLC analysis as kaempferol, apigenin, isorhamnetin, gallic acid, 4-hydroxybenzoic acid, caffeic acid, vanillic acid, syringic acid, ferulic acid, *p*-coumaric acid and sinapic acid. All these phenolics, except apigenin, were previously observed in shells and husks discarded during the production of sacha inchi oil [8]. Our results revealed that both leaf maturation and drying processes had a profound effect on the amounts of individual phenolics detected in sacha inchi leaves. The detected phenolics were higher in freeze-dried leaves than in oven-dried leaves. Our findings concurred with a previous study indicating that different phenolics possess distinct thermal stability [17]. Freeze-drying was previously reported as an effective drying process in terms of preserving thermolabile compounds in tea leaves, yielding a dried product with higher retention of phenolics compared with thermal drying processes [17]. However, even though TPCs of young and mature leaves dried under the same condition were similar in our study, both leaf types exhibited different predominant phenolics. Our findings were supported by previous studies. Nadeem and Zeb (2018) [18] observed significant changes in the amounts of phenolics during the maturation of fig leaves although the phenolic profiles detected in the leaves remained unchanged. Some studies reported variations in quantitative changes among plant phenolics over the maturation period. For instance, levels of gallic acid, 4-hydroxybenzoic acid and sinapic acid decreased with maturation, while levels of *p*-coumaric acid and syringic acid increased with maturation [18,19]. We found that *p*-coumaric acid, 4-hydroxybenzoic acid, ferulic acid and gallic acid were predominantly detected in young leaves regardless of the drying process. Similar results were observed in mature leaves, with the exception of gallic acid.

TPCs in sacha inchi leaves dried using the freeze-drying process were remarkably higher than those dried using the oven-drying process, regardless of leaf maturity. Similar results were reported by Sirichai et al. (2022) [20]. They investigated the effect of drying processes on phenolics in 10 different plants and found that TPCs in freeze-dried plants were 1.0–3.4 times higher than TPCs in oven-dried plants. Moreover, it has been demonstrated in the literature that the freeze-drying process could effectively preserve phenolics in plants, while TPCs in plant products obtained from freeze-drying process were close to those of the fresh materials [17]. TPCs in our freeze-dried leaf samples were comparable to those in fresh leaves of sacha inchi (~21 mg GAE/g DW) [12], and also far greater than previously reported TPCs in other parts of the plant [2,8]. These findings suggested that the freeze-drying technique could be adopted for retaining phenolic compounds with bioactivities from fresh sacha inchi leaves for further use. The highest TPC value obtained from freeze-dried mature sacha inchi leaves (~21 mg GAE/g DW) was low compared to leaves with notably high TPCs such as peppermint and tea leaves (approximately 94–202 [21] and 77–178 [17] mg GAE/g DW, respectively). Higher levels of phenolics released from plant cells were achieved by optimizing the extraction parameters, particularly solvent, temperature and time [8]. Research suggested that an increase in maturity resulted in higher TPC in sacha inchi leaves, as demonstrated by Mokhtar et al. (2021) [19]. A decline in TPC on leaf maturation was also evident in the literature [18]. This discrepancy might be due to differences in phenolic profiles and in the ability to accumulate individual phenolics across plant species.

Preservation methods, including drying, can alter the natural antioxidant capacity of plant-based food products; hence, the impact of such processes should be considered for the accurate evaluation of product health benefits. Phenolics are well recognized as the major contributor to the antioxidant activities of plants. A strong correlation between TPCs and antioxidant activities was reported in our previous research [20], which did not fully concur with the results from this study, suggesting that TPCs and antioxidant activities of sacha inchi leaves did not follow the same trend. Apart from phenolics, existing evidence demonstrated the presence of powerful non-phenolic antioxidants such as terpenoids, saponins and polysaccharides in sacha inchi leaves [10,11,12] as a possible explanation for the discrepancy between our study results and previous findings. Contrary to the findings from TPC determination, thermal drying using an oven had a positive effect on the antioxidant activities of young sacha inchi leaves as determined by DPPH radical-scavenging, ORAC and FRAP assays. The resulting data were clarified by the formation of non-phenolic antioxidants and/or stable intermediate products of carbohydrate caramelization and/or the Maillard reaction [22,23], when the studied oven-drying process (60 °C for 24 h) was applied to young leaves. Interestingly, the ORAC values indicated that the antioxidant potential of mature leaves was significantly higher with freeze-drying than with oven-drying, possibly due to differences in physicochemical properties (e.g., stomatal sensitivity and density, leaf surface area and polyphenol oxidase activity) [24,25], and the phenolic composition between young and mature leaves as aforementioned. Nevertheless, FRAP and ORAC values of freeze-dried leaves indicated that mature leaves exhibited higher antioxidant activities than young leaves, related to the results of TPCs. Our findings suggested that antioxidants in mature leaves were more susceptible to thermal processing than those in young leaves. However, different thermal treatments might produce diverse results [20] making this presumption valid for this study only. When extracted with water, ORAC values ranged from ~132 to ~164 μmol TE/g and showed comparable antioxidant potential to some common medicinal plants such as chicory, nettle, birch and laurel leaves (ORAC values of 132, 141, 142 and 170 μmol TE/g, respectively) [21].

The anti-glycation capacity of plant materials is often related to their phenolic compositions and antioxidant activities. A literature review on the inhibitory effect of phenolics on advanced glycation end products (AGEs) [26] suggested that phenolics detected in sacha inchi leaves inhibited glycation reactions with varying degrees of inhibitions and mechanisms. Phenolics usually retard the early stage of glycation but some (e.g., chlorogenic acid, sinapic acid, ferulic acid, vanillic acid and syringic acid) inhibit AGE formation in the advanced phase of glycation and also the subsequent crosslinking of proteins [27]. In addition to phenolics, certain non-phenolic compounds previously detected in sacha inchi leaves such as terpenoids and polysaccharides are capable of suppressing glycation reactions [28]. Our results suggested that infusions of young and mature leaves strongly inhibited D-glucose- and MG-mediated glycation reactions (>78% inhibition using extract concentration of 2.5 mg/mL), suggesting that these extracts mitigated cell deterioration and other complications associated with non-enzymatic glycation. Moreover, we also found that the anti-glycation properties of the extracts were positively influenced by the oven-drying process. Despite limited information on the effects of thermal processing on anti-glycation activity, a recent study on Chilean currant phenolics demonstrated that phenolic compounds under thermal treatment acted as anti-glycation agents [29]. The study also described the mechanisms of the thermal treatment that led to a decrease in AGE levels and lowering of electrophiles generated by a gastric digestion model [29].

Control of the key enzymes ameliorating the incidence of NCDs has attracted increasing attention in recent years due to the specific characterization of enzyme-substrate interactions. In this research, in vitro inhibitions of the enzymes relevant to some NCDs utilizing infusions of sacha inchi leaves were investigated and reported. Despite relatively poor inhibitions, the leaf infusions exhibited inhibitory activities against all the enzymes examined, with the exception of BACE-1. The low enzyme inhibitory activities observed possibly resulted from the thermal degradation of enzyme inhibitors during infusion and low concentrations of the leaf infusions in the reaction mixtures (final concentrations of 2.0–2.5 mg/mL). Therefore, we speculated that optimization of extraction conditions and/or increase in extract concentration would confer superior inhibitory activities. The inhibitory activities of sacha inchi leaf infusions were attributed to their phenolic contents since many plant phenolics act as enzyme inhibitors. Kaempferol, the most abundant flavonoid detected in the samples, exhibited strong inhibitory activities against AChE and BChE, as indicated by its IC_50_ of 92.8 and 71.0 µM, respectively [30]. This flavonoid also inhibited α-amylase, α-glucosidase [31] and lipase [32]. Likewise, *p*-coumaric acid and ferulic acid, the predominant phenolic acids in our samples, were previously reported to be inhibitors for carbohydrate- and lipid-hydrolyzing enzymes (IC_50_ for α-amylase of >30 and 9.5 mM, respectively, IC_50_ for α-glucosidase of >30 and 4.9 mM, respectively [33], and IC_50_ for lipase of 170.2 and 123.9 µM, respectively [34]). Furthermore, *p*-coumaric acid and ferulic acid retarded the activities of cholinesterases, and their inhibitions against BChE were much higher than their inhibitions against AChE [35]. Other phenolics detected in this study such as gallic acid, 4-hydroxybenzoic acid, caffeic acid and sinapic acid possessed inhibitory properties against the tested enzymes [35,36,37]. BACE-1 inhibitors (e.g., *p*-coumaric acid and gallic acid [38]) were present in all the samples but no BACE-1 inhibitory activities were observed because concentrations of these compounds in the leaf infusions were insufficient to produce the BACE-1 inhibitory effect. Overall, our results suggested that mature leaf infusions exhibited higher enzyme inhibitory activities compared to young leaves although the difference was small. This finding concurred with observations for TPCs, as discussed above. Interestingly, the drying processes employed in this study had minimal impacts on the inhibitory potential of sacha inchi leaf infusions, concurring with Sirichai et al. (2022) [20]. Several mechanisms were proposed to explain the inhibition of enzymes by bioactive compounds from plant origin, highlighting the importance of phytochemical compositions and the structural features of inhibitor candidates responsible for the observed inhibitory effects [39]. When present in crude biological extracts, many enzyme inhibitors can interact with metal ions and macromolecules such as polysaccharides and proteins, thus modifying their inhibition efficacies [40,41]. Synergism and/or antagonism among phenolics may occur and lead to remarkable changes in the resulting inhibitory capacity [35]. The inhibition of disease-associated enzymes by plant extracts is usually explained by the presence of phenolics but the inhibitory effects of non-phenolic substances should also be taken into consideration for a better understanding of the inhibitory actions of the extracts on enzymes. 

## 4. Materials and Methods

### 4.1. Sample Collection, Preparation, and Extraction

**Figure 1:**
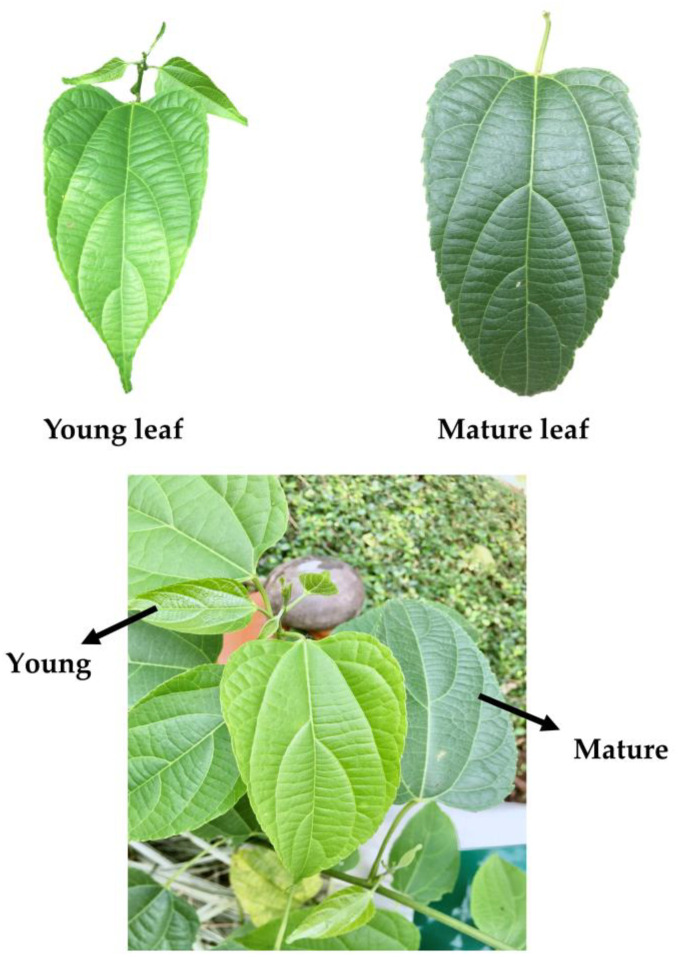
The physical appearances of young and mature leaves of sacha inchi.

Young and mature leaves of sacha inchi were obtained from Thai Rubber Land and Plantation Co., Ltd., Muang district, Chiang Rai Province, Thailand with the physical appearances as shown in Figure 1. According to Chumanee and Khoomsab (2020), the five topmost leaves with a light green color are considered as young leaves, and the dark green leaves appeared below the tenth leaves (counting from the top) are classified as mature leaves [42]. Our study had somewhat followed this verification, in which young leaves were selected from the 1st–5th leaves from the tip, while mature leaves were selected from the 6th–15th leaves. The plant samples, including young and mature leaves, were cleaned and separated into two groups. One was freeze-dried at −50 °C under a pressure of 0.086 mbar for 72 h using a Heto powerdry PL9000 freeze dryer from Heto Lab Equipment (Allerod, Denmark). The other was dried in a 60 °C/12 kW/380 V hot air oven from Kluay Num Thai (Bangkok, Thailand) for 5 h. The dry samples were ground into fine powder using a high-speed Cyclotec 1093 miller from Foss Tecator (Höganäs, Sweden). The moisture contents of powdery samples were determined using a Halogen HE53 moisture analyzer from Mettler–Toledo AG (Greifensee, Switzerland), and reported as percentage of moisture content. The color was also analyzed using a ColorFlex EZ spectrophotometer from Hunter Associates Laboratory (Reston, VA, USA), and expressed in terms of CIELAB units (L*, a*, b*). The moisture contents and color values are shown in Table S1. All samples were vacuum-packed in aluminum foil bags and stored at −20 °C until analysis. 

Sample extraction was performed as a tea infusion following Sirichai et al. (2022) [43]. The samples (1 g DW) were mixed with deionized water (100 mL), and incubated in a 95 °C WNE45 water bath shaker from Memmert GmBh (Eagle, WI, USA) for 5 min. A supernatant was collected after centrifugation at 3800× *g* for 10 min using a ROTINA 38R refrigerated centrifuge fromAndreas Hettich GmbH (Tuttlingen, Germany), and subsequently filtered through 0.45 µM polyethersulfone membranes. A tea infusion was kept at −20 °C until further analysis.

### 4.2. Determination of Phenolic Profiles

Phenolic profiles of all samples were determined using HPLC analysis following a well-established protocol of Temviriyanukul et al. (2020) with reliable validation as previously reported [44]. Briefly, the powdery samples were hydrolyzed under acidic methanol condition before loading into a 4.6 × 150 mm, 5 µm ZORBAX Eclipse XDB-C18 column attached to an HPLC system with photodiode array detection (Agilent Technologies, Santa Clara, CA, USA). The HPLC conditions included a flow rate of 0.6 m/min and a gradient solvent system as indicated in Table 4.

The authentic standards of phenolics including hesperidin (>90.0% HPLC, T), apigenin (>98.0% HPLC), quercetin (>98.0% HPLC, E), chlorogenic acid (>98.0% HPLC, T), 4-hydroxybenzoic acid (>99.0% GC, T), caffeic acid (>98.0% HPLC, T), ferulic acid (>98.0% GC, T), luteolin (>98.0% HPLC), naringenin (>93.0% HPLC, T), *p*-coumaric acid (>98.0% GC, T), kaempferol (>97.0% HPLC), myricetin (>97.0% HPLC), sinapic acid (>99.0% GC, T), and syringic acid (>97.0% T) were purchased from Tokyo Chemical Industry (Tokyo, Japan). Vanillic acid (≥97% HPLC) and gallic acid (97.5–102.5% T) were from Sigma-Aldrich (St. Louis, MO, USA), and isorhamnetin (≥99.0% HPLC) was from Extrasynthese (Genay, France). The detections at 338 nm and 368 nm were for identification of flavonoids, while phenolic acids were identified at 280 nm and 325 nm. Due to similarity of HPLC patterns between young and mature leaves (although possessing different quantities), only HPLC chromatograms of young leaves prepared by freeze-dried and oven-dried processes were shown in Figure S1. 

TPCs were determined using Folin–Ciocalteu’s phenol reagent according to the previously reported protocol [45]. Reactions were monitored on a 96-well UV/visible microplate reader (Synergy^TM^ HT, BioTek Instruments, Inc., Winooski, VT, USA) with a Gen 5 data analysis software at 765 nm. Gallic acid was used as a standard, and the results were expressed as mg GAE/g DW.

### 4.3. Determination of Antioxidant Activities

Antioxidant activities were determined spectrophotometrically by DPPH radical scavenging, FRAP, and ORAC assays using the well-established protocols as previously described by Sripum et al. (2017) [46] without any modifications. Trolox was used as a standard. All chemicals and reagents were purchased from Sigma-Aldrich (St. Louis, MO, USA). The antioxidant activity assays were performed on the SynergyTM HT 96-well UV-visible microplate reader, and the results were expressed as µmol TE/g DW.

### 4.4. Determination of Inhibitory Activities

Inhibitory activities against the key enzymes related to diabetes (α-amylase and α-glucosidase), obesity (lipase) and AD (AChE, BChE and BACE-1) using the well-established protocols as previously described by Sirichai et al. (2022) [20] without any modifications. Briefly, the enzyme reaction composed of enzyme, substrate, and indicator (if any) and kinetically detected at particular wavelength as shown in Table 5.

The inhibitory activity of BACE-1 was determined using BACE-1 assay kit from Sigma-Aldrich (St. Louis, MO, USA). The reaction was detected as end-pointed assay at an excitation wavelength (λ_ex_) of 320 nm and an emission wavelength (λ_em_) of 405 nm. The glycation reactions were detected using BSA induced by either MG or D-glucose as previously described [20]. The reaction was detected as end-pointed assay at λ_ex_ 330 nm and λ_em_ 410 nm. All chemicals and reagents were purchased from Sigma-Aldrich. The inhibitory assays were performed on the Synergy^TM^ HT 96-well UV-visible microplate reader, and the results were calculated as percentage of inhibition at particular extract concentrations using the following equation:

% Inhibition = (1 − (*B* − *b*)/(*A* − *a*)) × 100, (1)

where *A* is the initial velocity of the control reaction with enzyme, *a* is the initial velocity of the control reaction without enzyme, *B* is the initial velocity of the enzymatic reaction with the sample, and *b* is the initial velocity of the reaction with the sample but without enzyme.

### 4.5. Statistical Analysis

All experiments were conducted in triplicate (*n* = 3), and the results were presented as mean ± standard deviation (SD). Unpaired *t*-test was used to compare significant differences between values of two data sets. One-way analysis of variance (ANOVA) followed by Duncan’s multiple comparison test was employed to compare significant differences between values of more than two data sets. Values were considered significantly different when *p* < 0.05. All data were analyzed using the statistical package for the social sciences (version 18 for Windows, SPSS Inc., Chicago, IL, USA).

## 5. Conclusions

Products from seeds and oil of sacha inchi such as protein-rich seed powder and omega-3-rich oil have been industrialized and permitted for use in the United States and European countries as food ingredients, despite having no application of sacha inchi leaves in the food-related industry. Our results revealed that sacha inchi leaves contain several phenolics, which were quantitatively altered during the maturation and drying processes. Leaves prepared as infusions possess potential health benefits, including lowering the risk of oxidative stress-related disorders, inhibiting the key enzymes relevant to some NCDs (diabetes, obesity and AD) and retarding the aging process. Both young and mature leaves of sacha inchi showed promise as a source of bioactive compounds for health-related applications. Hot-water extraction was selected for easy and convenient preparation. Information obtained from this study will be useful for the future development of food/dietary supplement products from sacha inchi plant material, and also increase the commercial value of sacha inchi leaves by providing a wider variety of products to consumers. Since hot-water extraction as a tea infusion was chosen as a model for preparation of sacha inchi leaf extracts in this study, our leaf infusions have great potential for commercialization as a functional drink with further research in product development. Furthermore, sacha inchi leaf extract may be concentrated and used as a complementary food supplement, thus helping to enhance bioactivities of the food products. Other than tea and concentrated leaf extract, whole leaves may be consumed raw in fresh-cut salads or added to cooked recipes. However, further in vivo research is required to confirm our in vitro findings. The aspects of effective dose, bioavailability and pharmacokinetics should also be considered.

## Figures and Tables

**Table 1:** Phenolic compositions and total phenolic contents (TPCs) in young and mature sacha inchi leaves dried using freeze-drying and oven-drying processes.

Phenolics (mg/100 g DW)	Sacha Inchi Leaves			
	Young		Mature	
	Freeze-Dried	Oven-Dried	Freeze-Dried	Oven-Dried
**Flavonoids**				
Kaempferol	10.76 ± 0.13 ^e,†,^*	8.50 ± 0.18 ^f, §^	9.52 ± 0.15 ^f,^*	7.62 ± 0.06 ^e^
Apigenin	5.68 ± 0.16 ^f,^*	2.47 ± 0.06 ^g, §^	5.82 ± 0.12 ^g,^*	4.27 ± 0.16 ^f^
Isorhamnetin	1.52 ± 0.13 ^f,†,^*	1.22 ± 0.15 ^g, §^	2.80 ± 0.07 ^h,i,^*	0.77 ± 0.04 ^g^
**Phenolic acids**				
Gallic acid	43.25 ± 1.85 ^c,†,^*	32.91 ± 0.66 ^c,§^	21.48 ± 0.55 ^c^	20.21 ± 1.23 ^d^
4-Hydroxybenzoic acid	75.43 ± 3.63 ^b,†,^*	53.42 ± 2.53 ^b,§^	49.09 ± 1.39 ^b,^*	43.34 ± 1.55 ^b^
Vanillic acid	4.17 ± 0.15 ^f,†,^*	3.67 ± 0.09 ^g,§^	4.72 ± 0.21 ^g,h,^*	4.16 ± 0.19 ^f^
Caffeic acid	15.74 ± 0.41 ^d,†,^*	9.98 ± 0.40 ^f,§^	19.10 ± 0.41 ^d,^*	7.48 ± 0.19 ^e^
Syringic acid	1.51 ± 0.26 ^f,†^	1.51 ± 0.20 ^g^	1.79 ± 0.04 ^I,^*	1.46 ± 0.08 ^g^
*p*-Coumaric acid	89.71 ± 4.06 ^a,^*	28.34 ± 1.46 ^d^	89.15 ± 1.47 ^a,^*	31.28 ± 0.57 ^c^
Ferulic acid	73.10 ± 1.46 ^b,†^	71.79 ± 5.26 ^a,§^	90.96 ± 2.37 ^a,^*	83.66 ± 1.96 ^a^
Sinapic acid	17.58 ± 0.66 ^d,†,^*	14.39 ± 0.55 ^e,§^	14.18 ± 0.06 ^e,^*	8.82 ± 0.18 ^e^
**TPCs (mg GAE/g DW)**	20.74 ± 0.30 ^†,^*	12.82 ± 0.23 ^§^	21.37 ± 0.24 *	13.10 ± 0.11

All data are shown as mean ± standard deviation (SD) of triplicate experiments (*n* = 3). DW: dry weight; GAE: gallic acid equivalent; lowercase letters indicate significantly different contents of different phenolics in the same sample at *p* < 0.05 using one-way analysis of variance (ANOVA) and Duncan’s multiple comparison test; ^†^ and ^§^ show significantly different contents of the same phenolic in freeze-dried and oven-dried leaves, respectively, harvested at different maturity stages at *p* < 0.05 using unpaired *t*-test; * indicates significantly different contents of the same phenolic in freeze-dried and oven-dried leaves harvested at the same maturity stage at *p* < 0.05 using unpaired *t*-test.

**Table 2:** Antioxidant activities of aqueous extracts of young and mature sacha inchi leaves dried using freeze-drying and oven-drying processes.

Antioxidant Activities (µmol TE/g DW)	Sacha Inchi Leaves			
	Young		Mature	
	Freeze-Dried	Oven-Dried	Freeze-Dried	Oven-Dried
DPPH radical scavenging activity	0.049 ± 0.001 *	0.050 ± 0.001	0.049 ± 0.001	0.051 ± 0.002
FRAP activity	43.04 ± 3.51 ^†,^*	48.26 ± 2.21 ^§^	46.61 ± 3.87	45.70 ± 2.73
ORAC activity	131.58 ± 10.48 ^†,^*	163.93 ± 15.20 ^§^	162.20 ± 11.46 *	144.23 ± 17.34

All data are shown as mean ± standard deviation (SD) of triplicate experiments (*n* = 3). DW: dry weight; TE: Trolox equivalent; DPPH: 2,2–diphenyl–1–picrylhydrazyl; FRA: ferric ion reducing antioxidant power; ORAC: oxygen radical absorbance capacity; ^†^ and ^§^ show significantly different activities determined by the same antioxidant assay of freeze-dried and oven-dried leaves, respectively, at different maturity stages at *p* < 0.05 using unpaired *t*-test; * indicates significantly different activities determined by the same antioxidant assay between freeze-dried and oven-dried leaves of the same maturity stage at *p* < 0.05 using unpaired *t*-test.

**Table 3:** Enzyme inhibitory activities and anti-glycation activities of aqueous extracts prepared from dried leaves of sacha inchi.

Inhibitory Activities (% Inhibition)	Leaves of Sacha Inchi			
	Young		Mature	
	Freeze-Dried	Oven-Dried	Freeze-Dried	Oven-Dried
^1^ α-Amylase	37.13 ± 2.38 *	41.62 ± 3.74	39.68 ± 3.58	39.61 ± 2.21
^1^ α-Glucosidase	9.93 ± 0.94 ^†,^*	8.11 ± 0.65 ^§^	11.81 ± 1.19 *	9.96 ± 0.68
^2^ Lipase	9.49 ± 0.93	9.79 ± 0.79	9.43 ± 1.23	9.80 ± 1.14
^2^ AChE	4.15 ± 0.47 *	10.93 ± 1.21 ^§^	4.27 ± 0.69 *	14.64 ± 1.11
^2^ BChE	24.13 ± 1.97 ^†,^*	20.88 ± 1.27 ^§^	30.09 ± 1.62 *	25.36 ± 1.13
^2^ BACE-1	ND	ND	ND	ND
^1^ Glucose-induced glycation	78.30 ± 2.25 ^†,^*	82.12 ± 2.76 ^§^	83.98 ± 0.32 *	92.98 ± 2.40
^1^ MG-induced glycation	81.35 ± 0.54 ^†,^*	84.22 ± 0.23f ^§^	84.11 ± 1.97 *	90.51 ± 1.85

All data are shown as mean ± standard deviation (SD) of triplicate experiments (*n* = 3). AChE: acetylcholinesterase; BChE: butyrylcholinesterase; BACE-1: β-secretase; MG: methylglyoxal; ND: not detected; ^†^ and ^§^ show significantly different inhibitory activities determined by the same assay of freeze-dried and oven-dried extracted leaves at different maturity stages, respectively, at *p* < 0.05 using unpaired *t*-test.; * indicates significantly different inhibitory activities determined by the same assay between freeze-dried and oven-dried extracted leaves of the same maturity stage at *p* < 0.05 using unpaired *t*-test.; a positive control of each assay was performed on the reaction using the enzyme without the extract; ^1^ final concentration of the extracts was 2.5 mg/mL; ^2^ final concentration of the extracts was 2.0 mg/mL.

**Table 4:** A gradient solvent system of high-performance liquid chromatography (HPLC).

Time (min)	Solvent A (%)	Solvent B (%)	Solvent C (%)
0	90	6	4
5	85	9	6
30	71	17.4	11.6
60	0	85	15
61	90	6	4
66	90	6	4

Solvent A = Milli-Q water (18.2 MΩ.cm resistivity at 25 °C) containing 0.05% (*v/v*) trifluoroacetic acid (TFA); solvent B = methanol containing 0.05% (*v/v*) TFA; solvent C = acetonitrile containing 0.05% (*v/v*) TFA.

**Table 5:** The compositions and detecting wavelengths of enzyme reactions.

Enzyme	Substrate	Indicator	Detected wavelength
≥10 unit/mg (type VII), porcine pancreatic α-amylase	*p*-nitrophenyl-α-d-maltopentaoside		405 nm
≥10 U/mg protein (type I), *Saccharomyces cerevisiae* α-glucosidase	*p*-nitrophenyl-α-d-glucopyranoside		
≥700 unit/mg (type VII), *Candida rugosa* lipase	DNPDB	DTNB	412 nm
1000 units/mg, *Electrophorus electricus* AChE	acetylthiocholine		
≥10 units/mg, equine serum BChE	butyrylthiocholine		

DNPDB: 5–5′-dithiobis (2-nitrobenzoic-*N*-phenacyl-4,5-dimethyyhiazolium bromide; DTNB: 5,5′-dithiobis (2-nitrobenzoic acid); AChE: acetylcholinesterase; BChE: butyrylcholinesterase.

## Data Availability

Data are contained within this article and supplementary materials.

## Supplementary Materials

The following supporting information can be downloaded at: https://www.mdpi.com/article/10.3390/plants11111515/s1, Table S1: Moisture contents and color values of young and mature sacha inchi leaves dried using freeze-drying and oven-drying processes.; Figure S1: Examples of HPLC chromatograms of the phenolics identified in young leaves underwent (**A**) freeze-drying and (**B**) oven-drying processes.
