# Supplementary material for: Effects of Maturity and Thermal Treatment on Phenolic Profiles and In Vitro Health-Related Properties of Sacha Inchi Leaves
Source: Plants (Basel). 2022 Jun 5;11(11):1515. doi: 10.3390/plants11111515 (PMC9182973; doi:10.3390/plants11111515)
Supplement: Supplementary file 1 [file plants-11-01515-s001.zip › plants-1751147-supplementary (revised).pdf]

plants

MDPI

Supplementary materials

Effects of Maturity and Thermal Treatment on Phenolic Profiles and In Vitro Health-Related Properties of Sacha Inchi Leaves

Suwapat Kittibunchakul [1], Chatrapa Hudthagosol [2], Promluck Sanporkha [2], Suwimol Sapwarobol [3], Uthaiwan Suttisansanee [1] and Yuraporn Sahasakul [1,*]

[1] Food and Nutrition Academic and Research Cluster, Institute of Nutrition, Mahidol University, Salaya, Phuttamonthon, Nakhon Pathom 73170, Thailand; suwapat.kit@mahidol.ac.th (S.K.); uthaiwan.sut@mahidol.ac.th (U.S.); yuraporn.sah@mahidol.ac.th (Y.S.)

[2] Faculty of Public Health, Mahidol University, Ratchathewi, Bangkok 10400, Thailand; chatrapa.hud@mahidol.ac.th (C.H.); promluck.san@mahidol.ac.th (P.S.)

[3] Faculty of Allied Health Sciences, Chulalongkorn University, Pathumwan, Bangkok 10330, Thailand; suwimol.sa@chula.ac.th (S.S.)

* Correspondence: yuraporn.sah@mahidol.ac.th

Supplementary Table S1:

Moisture contents and color values of young and mature sacha inchi leaves dried using freeze-drying and oven-drying processes.

Measurements	Leaves of sacha inchi			
	Young		Mature	
	Freeze-dried	Oven-dried	Freeze-dried	Oven-dried
Moisture content (%)	5.67 ± 0.25 †,*	4.77 ± 0.06 §	6.30 ± 0.10 *	6.67 ± 0.15
L*	52.87 ± 0.01 †,*	46.66 ± 0.06 §	52.75 ± 0.03 *	48.04 ± 0.05
a*	-7.63 ± 0.01 †,*	-0.46 ± 0.01 §	-8.02 ± 0.01 *	-1.91 ± 0.04
b*	31.22 ± 0.02 †,*	25.73 ± 0.02 §	30.31 ± 0.01 *	22.86 ± 0.06

All data were expressed as mean ± standard deviation (SD) of triplicate experiments (n = 3). † and § show significantly different values of the same measurement obtained from freeze-dried and oven-dried leaves at different maturity stages, respectively.; * indicates significantly different values of the same measurement between freeze-dried and oven-dried leaves of the same maturity.; All comparisons were made at $p < 0.05$ using unpaired t-test.; L*, a*, and b* indicate relative lightness/darkness, redness/greenness, and yellowness/blueness of the samples, respectively. The percentage of moisture content in dried sacha inchi leaf samples ranged from 4.77 to 6.67 (Table S1). Significant differences in moisture content were found in both young and mature leaves dried using different processes, namely freeze-drying and oven-drying. Mature leaves possessed higher levels of moisture content than young leaves when dried using the same process. The CIELAB color values suggested that the method employed for drying significantly affected the three color parameters (L*, a* and b*) of the samples (Table S1). At the same maturity stage, freeze-dried leaves exhibited greater brightness and yellowness than oven-dried leaves, as it was indicated by the higher L* and b* values, respectively. All samples possessed negative a* values, and the negativity effect was more pronounced in freeze-dried leaves compared with oven-dried leaves, regardless of maturity status, thereby demonstrating higher degrees of greenness of the freeze-dried samples. Likewise, when using the same drying process, young and mature leaves showed slight differences in their L*, a* and b* values although the differences were not statistically significant. Our results showed that moisture content and color of the dried leaves were influenced by drying processes and the variables used. All leaf samples dried under the studied operating conditions (freeze-drying at -50 °C and 0.086 mbar for 72 h, and oven-drying at 60 °C for 24 h) possessed low moisture contents at less than 7%. Drying of herbs and medicinal plants to achieve the moisture content of 10% or below is generally recommended for avoiding biochemical changes and microbial contamination during long-term storage . Interestingly, we found that the stage of leaf maturity could affect the degree of water loss

from sacha inchi leaves via drying, as indicated by the higher residual moisture content in the dried mature leaves compared with the dried young leaves. This finding was in agreement with a study by Hopper et al. (2014) [1], in which leaf maturity was reported to influence the leaf dehydration response due mainly to differences in stomatal sensitivity and density, as well as in leaf size. Removing moisture from plant materials by drying could induce the color changes, and color characteristics of a dried plant product are varied, depending on the procedures and conditions used for drying, particularly the temperature. The effect of thermal treatment in the drying processes on plant color was observed in the present study. The measured L* values suggested that oven-drying process rendered all leaf samples darker color when compared with freeze-drying process. The increased darkness could be due to the formation of brown pigments through the activity of plant polyphenol oxidases, and the non-enzymatic browning reactions under mild heating conditions [2]. Likewise, chlorophylls, the major pigments in leaves, are converted to pheophytins at elevated temperatures, with consequent changes in color from bright green to dark olive . This could also be a likely explanation for the better retention of green color observed in freeze-dried samples, as indicated by a* values. Concerning b* parameter, thermal decomposition of compounds with yellow color might be the reason for the lower b* values (yellowness) in oven-dried leaves samples. Although it was not clearly indicated by our results, it was likely that the effect of drying processes on leaf color became less pronounced with increasing leaf maturity.

Supplementary Figure S1:

Examples of HPLC chromatograms of the phenolics identified in young leaves underwent (**A**) freeze-drying and (**B**) oven-drying processes.

(A)

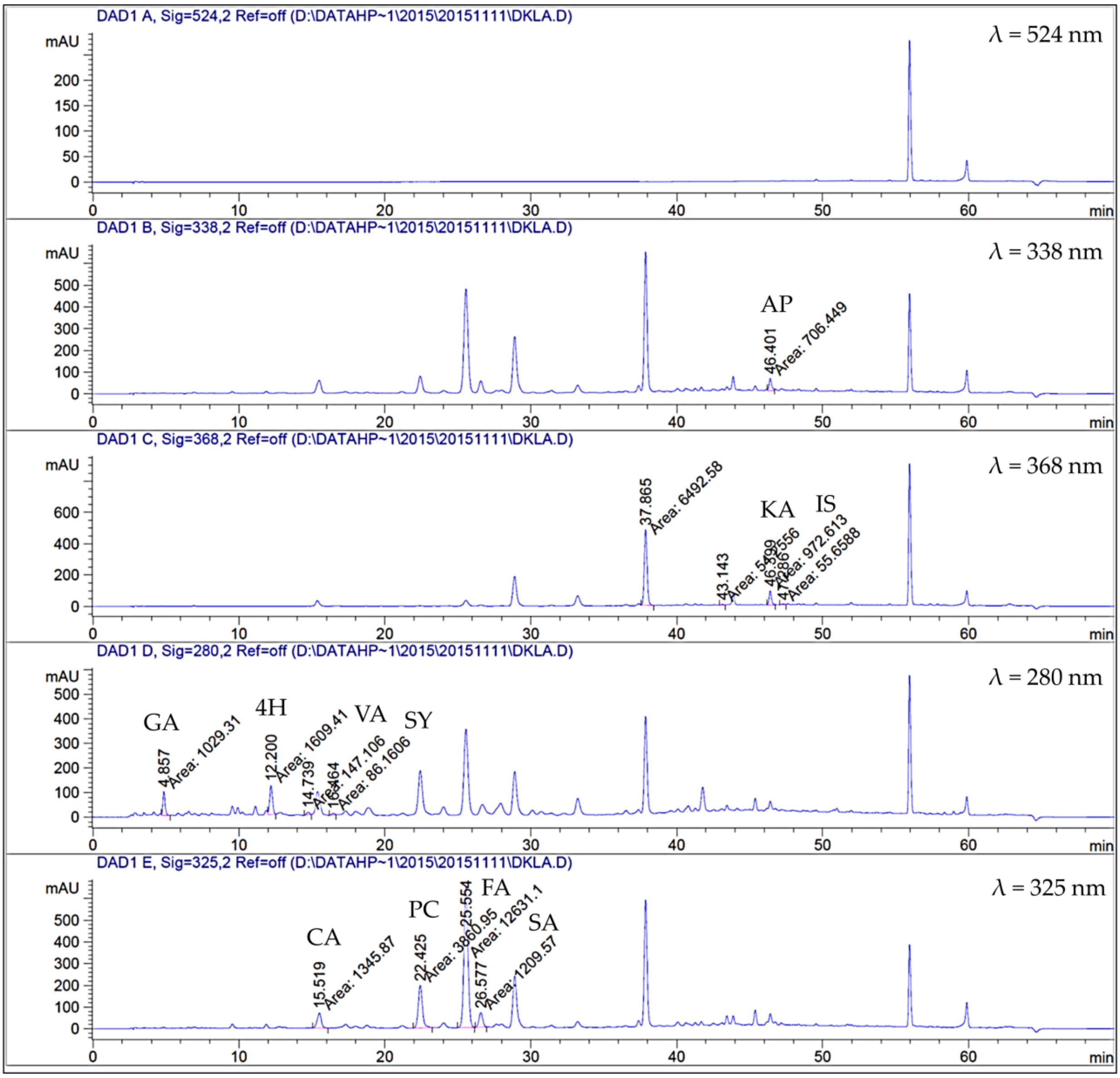

Peaks were identified with retention times (tR) compared with authentic standards, and confirmed the characteristic spectra by photodiode array.; AP: apigenin (tR = 46.41 ± 0.05 min); KA: kaempferol (tR = 46.23 ± 0.04 min); IS: isorhamnetin (tR = 46.94 ± 0.03 min); GA: gallic acid (tR = 4.85 ± 0.04 min); 4H: 4-hydroxybenzoic acid (tR = 12.21 ± 0.08 min); VA: vanillic acid (tR = 14.69 ± 0.08 min); SY: syringic acid (tR = 16.21 ± 0.08 min); CA: caffeic acid (tR = 15.50 ± 0.09 min); PC: *p*-coumaric acid (tR = 22.40 ± 0.11 min); FA: ferrulic acid (tR = 25.53 ± 0.11 min); SA: sinapic acid (tR = 26.46 ± 0.12 min).

Supplementary Figure S1 (Cont.):

Examples of HPLC chromatograms of the phenolics identified in young leaves underwent (**A**) freeze-drying and (**B**) oven-drying processes.

(B)

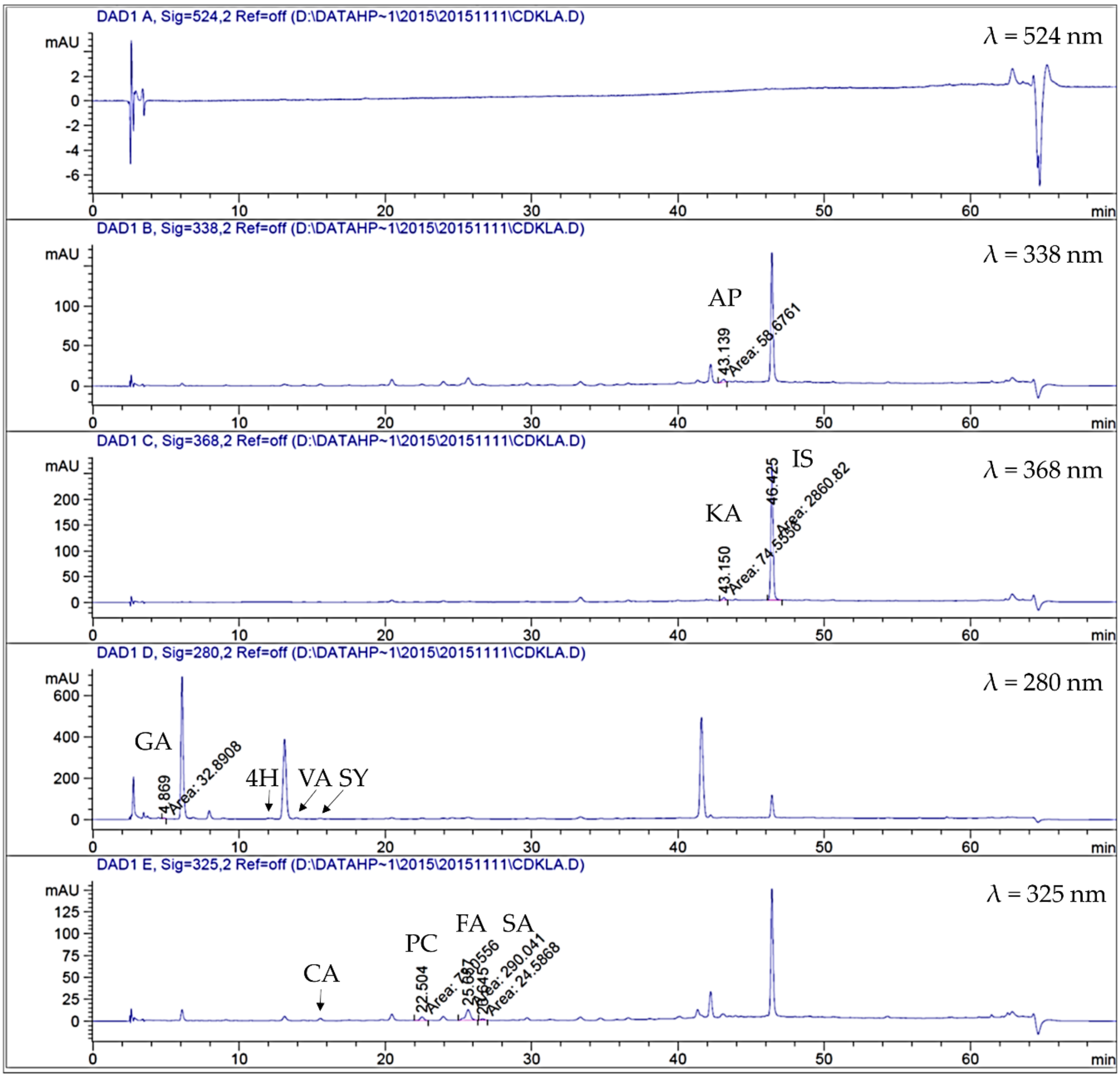

Peaks were identified with retention times (tR) compared with authentic standards, and confirmed the characteristic spectra by photodiode array.; AP: apigenin (tR = 46.41 ± 0.05 min); KA: kaempferol (tR = 46.23 ± 0.04 min); IS: isorhamnetin (tR = 46.94 ± 0.03 min); GA: gallic acid (tR = 4.85 ± 0.04 min); 4H: 4-hydroxybenzoic acid (tR = 12.21 ± 0.08 min); VA: vanillic acid (tR = 14.69 ± 0.08 min); SY: syringic acid (tR = 16.21 ± 0.08 min); CA: caffeic acid (tR = 15.50 ± 0.09 min); PC: *p*-coumaric acid (tR = 22.40 ± 0.11 min); FA: ferrulic acid (tR = 25.53 ± 0.11 min); SA: sinapic acid (tR = 26.46 ± 0.12 min).

References

1. Hopper, D.W.; Ghan, R.; Cramer, G.R. A rapid dehydration leaf assay reveals stomatal response differences in grapevine genotypes. *Hortic. Res.* **2014**, *1*, 2.
2. Hong, P.K.; Betti, M. Non-enzymatic browning reaction of glucosamine at mild conditions: Relationship between colour formation, radical scavenging activity and α-dicarbonyl compounds production. *Food Chem.* **2016**, *212*, 234-243, doi:10.1016/j.foodchem.2016.05.170.
